# Monitoring of Pb Exposure in Waterfowl Ten Years after a Mine Spill through the Use of Noninvasive Sampling

**DOI:** 10.1371/journal.pone.0057295

**Published:** 2013-02-20

**Authors:** Monica Martinez-Haro, Mark A. Taggart, Hugues Lefranc, Rosa C. Martín-Doimeadiós, Andy J. Green, Rafael Mateo

**Affiliations:** 1 Instituto de Investigación en Recursos Cinegéticos – IREC (CSIC-UCLM-JCCM), Ciudad Real, Spain; 2 Department of Wetland Ecology, Estación Biológica de Doñana – EBD (CSIC), Sevilla, Spain; 3 Environmental Research Institute (ERI), University of the Highlands and Islands, Thurso, Scotland; 4 Department of Analytical Chemistry and Food Technology, Faculty of Environmental Sciences and Biochemistry, University of Castilla-La Mancha, Toledo, Spain; University of Lethbridge, Canada

## Abstract

Lead exposure in waterfowl was studied using noninvasive fecal sampling in the Guadalquivir Marshes in Spain, an area affected by the 1998 Aznalcóllar mine disaster. Feces of greylag geese (*Anser anser*, n = 191) and purple gallinule (*Porphyrio porphyrio*, n = 91) were collected from three different impacted sites (Entremuros, Caracoles and Cerro de los Ánsares) during the winters of 2004 to 2008. Lead and aluminium (an indicator of sediment ingestion) and Pb isotope signatures (to discriminate between sources of Pb exposure) were analyzed in freeze-dried, acid digested samples. The concentrations of fecal porphyrins and biliverdin were determined as noninvasive biomarkers to study Pb exposure effects. Results showed a decrease in Pb exposure over time in wintering greylag geese. In contrast, for purple gallinule resident in the Entremuros a clear trend was not evident. For both species, sediment ingestion appeared to be the main source of exposure to Pb. In the Entremuros, some samples from purple gallinule were detected with higher Pb levels than expected for simple soil ingestion, and these had Pb isotopic profiles compatible with mining sludge or Pb shot. Whilst fecal Pb isotopic profiles were effective in differentiating between samples from sites with different levels and sources of pollution, the combined use of element ratios (such as Pb/Al) and other non-traditional stable isotope signatures may also prove worthwhile. Overall, the fecal Pb levels detected were below those described in feces for waterfowl from other uncontaminated areas(<10 µg/g d.w.). Despite this, for both species fecal Pb levels were positively correlated with porphyrin excretion, and for purple gallinule, with the coproporphyrin III/I ratio, suggesting some subtle effects on heme synthesis in birds. Ten years after the mine spill, Pb contamination in birds by this pollution source was still detectable and subtlethal effects may persist.

## Introduction

The Guadalquivir marshes are an extensive mosaic of wetlands of deltaic origin, located in south-western Spain. Due to its geographical location and environmental characteristics, these marshes are one of the most important wintering sites for migratory waterbirds in the Western Palearctic [1], [2]. The marshes are protected within the adjacent Doñana National and Natural Parks and Doñana Ramsar site, and the National Park is designated by UNESCO as a “World Heritage Site and Biosphere Reserve”.

In April 1998, the Guadalquivir marshes were severely affected by the Aznalcóllar mine accident [3], [4], when a tailing pond dike collapsed at the Los Frailes mine (located ∼45 km north of Doñana National Park). This mine was one of many that exploit the vast Iberian Pyrite Belt, one of the largest sulphide deposits in the world [5], [6]. The tailings spill made headlines around the world and released ∼5–6 million cubic meters of acidic and highly toxic waste containing high levels of heavy metals such as Zn, Pb, As and Cu [3], [4]. The spill flowed southward into the Guadiamar River, and continued for 45 km, reaching the edge of Doñana National Park. Much of the waste was finally retained using rapidly constructed temporary dams in a hydrologically confined wetland channel called the Entremuros (which means “between walls”), within the Doñana Natural Park [4], [7]. Initial estimates showed that >2,700 ha of the protected Doñana area had been contaminated by the spill, with the 900 ha Entremuros site most severely affected [3], [4]. In terms of Pb pollution specifically, concentrations up to 2,500 µg/L were recorded in open water, and 690 µg/g were detected in Doñana sediments after the spill [3]. Such levels greatly exceeded background concentrations previously recorded in the Doñana area and those in unaffected areas [3], [8]. Hence, immediately after the spill the Andalusian and Spanish authorities began to clean and restore the affected areas, undertaking preventive and mitigating measures [3], [4]. Nevertheless, low-level residual heavy metal contamination remained [9]–[13].

After the spill, sick and dead birds with high levels of heavy metals in tissues were found in Doñana National Park and surroundings [3], [7], [14]–[16]. Similarly, several studies have shown elevated concentrations of heavy metals in invertebrates and in macrophytes [17]–[19]. The Aznalcóllar accident was potentially very significant for waterbirds that depend on the impacted habitats, especially for those which also actively ingest sediment when feeding [20]. In polluted areas, this sediment ingestion route can be a very important heavy metal exposure pathway in birds [21]–[23]. A previous study addressed the effect of the Aznalcóllar spill on wintering greylag geese (*Anser anser*) during the 2001/2002 wintering season, analyzing the concentrations of various metals/metalloids (Pb, Zn, Cu, Mn and As) in feces from 5 different sites within the Guadalquivir marshes [24]. Results showed that the highest metal concentrations, especially for Pb, were in fecal samples from the Entremuros [24]. In addition, the study detected biological effects in geese due to exposure to the mining pollution, through the fecal analysis of biomarkers (such as porphyrins) [24].

Here, we aim to study trends over time in Pb exposure in wintering greylag geese and resident purple gallinule (*Porphyrio porphyrio*) in Doñana, using feces as noninvasive samples. These two bird species were amongst those most affected by the Aznalcóllar spill [16]. Fecal sampling is used to evaluate recent exposure and local contamination (e.g., [24], [25]). The Pb excreted by feces corresponds to that fraction ingested but not absorbed for the animal, and the mean retention time of ingested food/sediment (and thus most Pb, excluding large particles like Pb shot which could be retained in the gizzard as grit) in herbivorous waterfowl is around 2 h [26]–[29]. In addition to fecal Pb analysis, the relationship between fecal Pb and Al is presented alongside Pb isotopic signatures [25], [30]–[33]. The relationship between Pb and Al is used as an indicator of sediment ingestion [25], [32], [33], whilst Pb isotope signatures help to discriminate between different sources of Pb by identifying the geological origin of any given Pb in a sample [25], [30], [31]. Finally, fecal porphyrin and biliverdin profiles are used to detect potential toxicological effects/perturbations [34]. Lead exposure is commonly associated with impaired heme synthesis [35], [36] since it can inhibit the activity of enzymes such as δ–aminolevulinic acid dehydratase (ALAD), coproporphyrinogen oxidase and ferrochelatase [35]. Disruption of heme synthesis generates a surplus in the production of different heme precursors, which are then excreted at higher levels through urine or feces [35]. Biliverdin is a green bile pigment that comes from heme group catabolism. Lead poisoned birds developing hemolytic anemia frequently show green-stained urates, due to the increased excretion of biliverdin and higher concentrations of biliary biliverdin [37], [38].

## Materials and Methods

### Collection sites and field procedures

**Figure 1:**
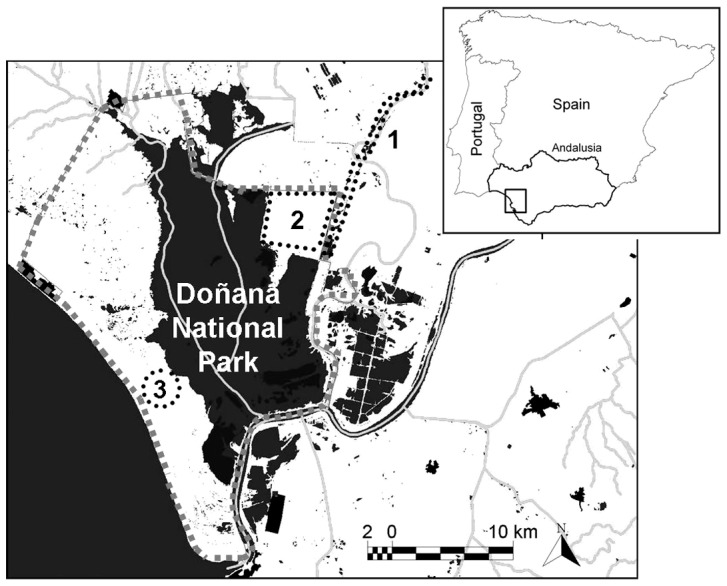
Map of the study area located in Andalusia (southern Spain) showing the limits of the Doñana National Park (grey line) and the three study sites (black dotted lines). 1 = Entremuros; 2 = Caracoles; 3 = Cerro de los Ánsares dune.

Three sites within Doñana were selected for this study (Fig. 1). The Entremuros was the site most affected by the spill. It lies within the Doñana Natural Park and represents an important refuge area for breeding birds and moulting waterfowl in the summer. It also acts as a habitat corridor, which separates the marshes of the National Park from the rice fields to the east. The Caracoles area (adjacent to Entremuros) was an agricultural area at the time of the spill, and was effectively isolated from the contaminated area by a dyke. As part of an extensive restoration project that followed the disaster, this area was restored as a wetland in 2004–2005 and then incorporated into the National Park [39]. Finally, the Cerro de los Ánsares dune, which is a mobile dune within the National Park, was also unaffected by the spill. However, it was heavily exploited by geese hunters up until 1983 (when hunting was banned in the park). This hunting activity has left a legacy of Pb shot pellets at this site, and 16.2 Pb shot pellets/ha were present in the upper 20 cm layer in 1997 [40]. Thus, this site represents another important source of Pb within the study area, since geese commonly ingest gizzard grit (and therefore shot) within these dunes [40]–[42].

Wintering geese arrive in October to Guadalquivir marshes and stay until late February or early March. Fecal sampling took place during four winter seasons (December – February) in successive years from 2004 to 2008 (Table 1). Feces of greylag geese were collected after flock identification with binoculars or a telescope from the edge of the Entremuros, Caracoles and Cerro de los Ánsares. No samples were collected from Entremuros after 2005/2006 because no geese flocks were located in that area during our field visits. Likewise, fecal samples for purple gallinule were only collected from the Entremuros since this species was absent from the other two study sites. Only fresh excrement was collected, and samples were taken at a minimum distance of two meters apart in order to reduce the likelihood of taking multiple samples from one individual. Each excreta was picked up carefully, adhered sediment was removed, and the sample was placed into a zip-lock bag and subsequently frozen at −20 °C until analysis. Sediment samples (0–5 cm) were taken in 2008 from the Entremuros and Caracoles study sites.

**Table 1:** Geometric mean (range) of Pb and Al concentrations (in µg/g dry weight) in greylag geese and purple gallinule feces, and samples of sediment collected at different study sites in Doñana during the 2004 to 2008 winters.

	Site	Season	N	Pb (µg/g d.w.)	Al (µg/g d.w.)
Feces					
Greylag geese	Entremuros	01/02*	60	14.99^A^ ‡ (5.10–42.90)	2336 (302–9125)
		04/05	30	4.45^B^ (1.05–48.96)	2268 (nd^†^–29749)
		05/06	30	2.98^B^ (0.93–13.77)	1568 (nd–12922)
	Caracoles	04/05	30	4.61^A^ (1.15–19.97)	5364 (5750–18432)
		05/06	30	3.14^AB^ (0.67–15.41)	2673 (nd–13341)
		06/07	20	2.16^BC^ (0.63–7.79)	994 (nd–11236)
		07/08	31	2.21^C^ (0.57–25.25)	2688 (nd–56452)
	Cerro	05/06	20	5.22 (2.04–12.52)	5073 (1496–12200)
Purple gallinule	Entremuros	04/05	29	2.59^B^ (nd–6.13)	2134 (193–7759)
		05/06	32	4.07^B^ (1.25–319.54)	5339 (1364–23548)
		07/08	30	6.02^A^ (nd–114.06)	1960 (329–5157)
Sediment					
	Entremuros	08	9	52.57^A^ (40.15–76.37)	47814 (36063–54676)
	Caracoles	08	26	40.86^B^ (27.06–63.26)	38587 (30343–45470)

Data from 2001/2002 previously described [24] are included.

* Data from [24]; ^†^nd = below detection limit.

‡ Means sharing a superscript letter were not significantly different among seasons for each locality for feces and between sites for sediments (p>0.05).

All necessary permits were obtained for the field studies. Permits required to enter Doñana National Park were granted by the Consejería de Medio Ambiente, Junta de Andalucía.

### Sample analysis

Feces (n = 282) and sediment (n = 35) samples (0.2–0.3 g) were freeze dried, acid digested and analyzed using graphite furnace (Pb) and nitrous oxide-acetylene flame (Al) atomic absorption spectroscopy (AAnalyst800, PerkinElmer), following methods described previously [32]. Blanks, a certified soil reference material (GBW07406), and certified bush, branches and leaves reference material (NCS DC 73349), were also processed in each batch of digestions (to provide quality control data). Limits of detection (LODs) by dry weight were 0.28 µg/g for Pb and 88.80 µg/g for Al. Mean percentage Pb recoveries(±%RSE) were 106.9%(±3.4%, n = 8) for soil and 99.5%(±3.5%, n = 9) for the bush, branches and leaves CRM. For Al, the mean% recovery(±%RSE) for the bush, branches and leaves CRM was 100.4%(±1.69%, n = 6).

In a sub-selection of the acid digested feces (n = 72), the stable isotopes ^206^Pb, ^207^Pb, and ^208^Pb were also analyzed by inductively coupled plasma mass spectrometry [33]. A certified NIST Pb isotope standard was used (SRM 981), which has an isotopic composition (mean±95%) of 24.1442±0.0057% for ^206^Pb, of 22.0833±0.0027% for ^207^Pb, and of 52.3470±0.0086% for ^208^Pb. All isotope ratios determined for SRM 981 during analysis were within 1% of the certified value (before a nominal rolling correction was applied to all data).

Finally, another sub-selection of feces (n = 71) was used for porphyrin and biliverdin analysis. This was conducted using liquid chromatography single quadrupole mass spectrometry (LC/MS) [33]. The recovery for the extraction procedure used was calculated with fecal samples (n = 6) spiked with porphyrins and biliverdin. Mean % recoveries(±%RSD) for coproporphyrin I, III, mesoporphyrin IX, protoporphyrin IX, and biliverdin were 107±8%, 73±8%, 51±8%, 18±2% and 23±6% respectively.

### Statistical analysis

Where necessary, data were log-transformed prior to statistical analysis to meet parametric assumptions. Data were analyzed using General Lineal Models (GLMs) considering Pb or Al concentrations, Pb isotopes signatures, and porphyrin and biliverdin concentrations as dependent variables; sampling site, season and/or species as factors; and Al or Pb concentration as covariates. Lead and Al concentrations in feces of greylag geese collected in the Entremuros during the 2001/2002 sampling season (n = 60; from [24]) were also compared with data from 2004/2005 and 2005/2006 in the Entremuros. Post-hoc differences were studied with Tukey tests. Additionally, when necessary, marginal means obtained with the models were compared using the least significant difference (LSD) test. Pearson correlations were used to study the relationships between dependent variables. As porphyrins and biliverdin have a common biliary origin in bird excreta, for GLMs fecal porphyrin concentrations were expressed relative to biliverdin (as the ratio porphyrin/biliverdin) in order to compensate for variation in the amount of bile or feces excreted due to dietary variations [24]. All tests were performed using SPSS 19.0, with the level of statistical significance set at p≤0.05.

## Results

### Sediment analyses

Mean Pb and Al concentrations were higher in sediment samples from the Entremuros than from Caracoles (Pb: F_1,33_ = 9.85, p = 0.004; Al: F_1,33_ = 22.72, p<0.001; Table 1). In the Entremuros, a positive relationship between Pb and Al levels was found in sediments (r = 0.813, p = 0.008, n = 9), but this was not the case in Caracoles (r = −0.378, p = 0.057, n = 26).

### Fecal analyses

**Figure 2:**
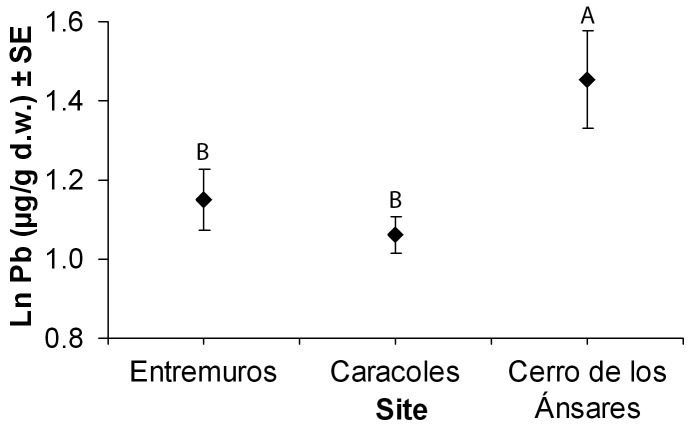
Estimated marginal means(±SE) of fecal Pb concentrations for greylag geese by sampling site as obtained in a GLM model when considering Al as a covariable. Means sharing a capital letter did not differ significantly (LSD, p>0.05).

Mean Pb levels in feces of greylag geese differed among sites (F_2,184_ = 4.63, p = 0.011; Fig. 2) and between winters (F_3,184_ = 7.74, p<0.001). Mean Pb levels showed a positive relationship with Al (F_1,184_ = 309.86, p<0.001). Higher mean Pb concentrations were detected in fecal samples from Cerro de los Ánsares than from Entemuros and Caracoles (Fig. 2). For feces of geese collected in the Entremuros, a significant decrease in Pb level was detected over the period 2001/2002 to 2004/2005 and 2005/2006 (geometric means 15, 4.5 and 3.0 µg/g, respectively; F_2,116_ = 358.03, p<0.001; Table 1).

**Figure 3:**
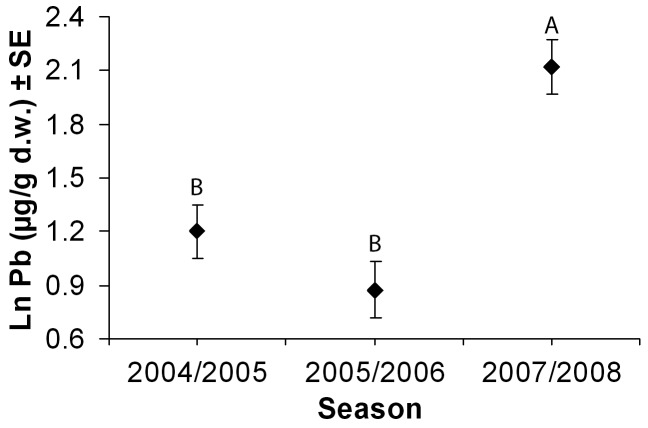
Estimated marginal means (± SE) of fecal Pb concentrations for purple gallinule at Entremuros by sampling season as obtained in a GLM model when considering Al as a covariable. Means sharing a capital letter did not differ significantly (LSD, p>0.05).

Purple gallinule feces differed in terms of Pb concentrations between winters (F_2,87_ = 10.29, p<0.001; Table 1; Fig. 3), but a clear trend was not evident (i.e., levels were similar in 2004/2005 and 2005/2006, then increased in 2007/2008). In addition, Pb in purple gallinule feces showed a positive relationship with Al (F_1,87_ = 21.39, p<0.001).

**Figure 4:**
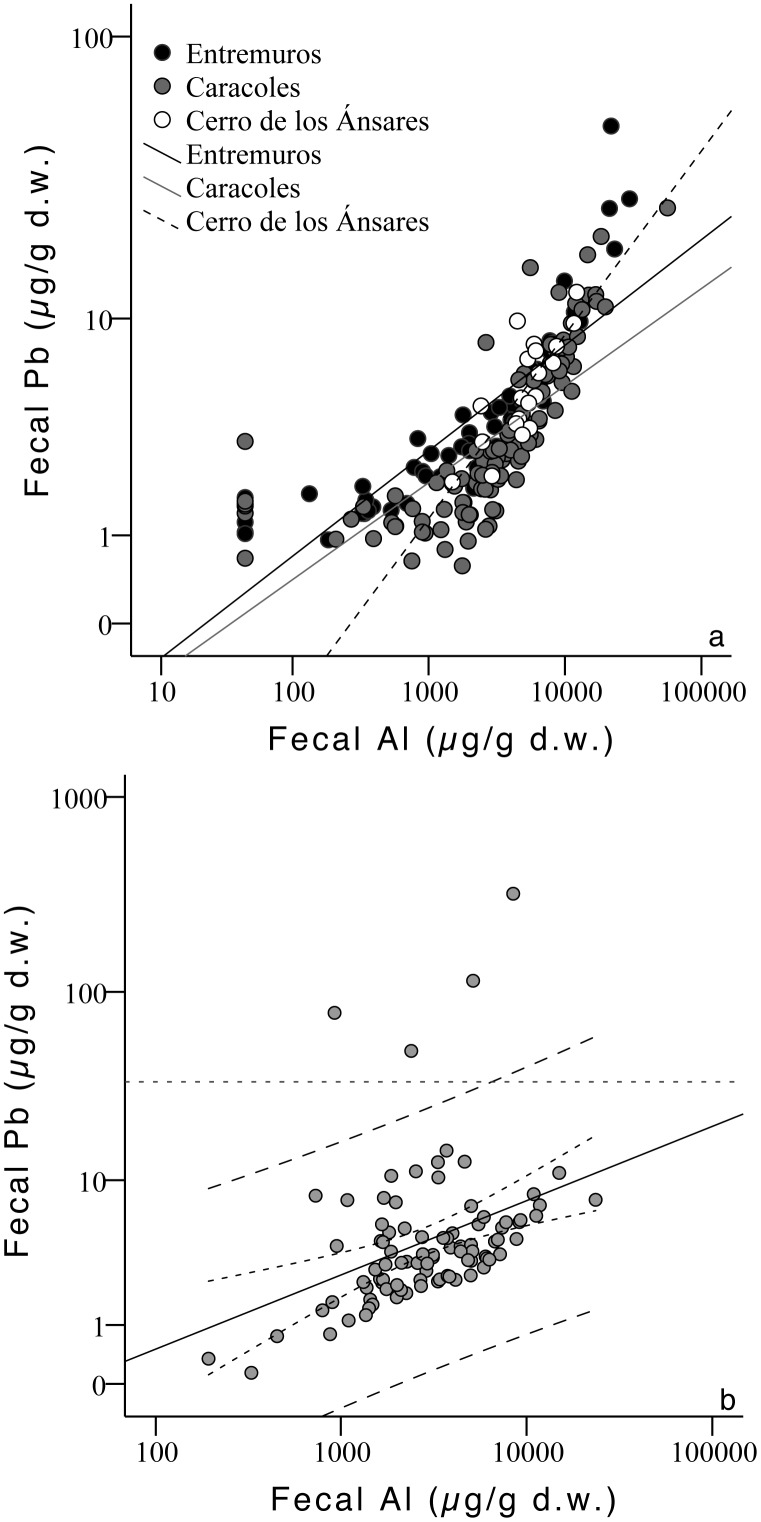
Relationship between Pb and Al concentration (μg/g dry weight) in (a) feces of greylag geese from three different sampling sites, and (b) feces of purple gallinule collected in Entremuros (black dotted lines represent the 95% confidence and prediction intervals and the horizontal control line shows the 34 μg/g Pb level indicative of exposure to point sources of Pb; [32], [33]).

For both species, fecal Pb levels were positively and significantly correlated with Al in all sites (greylag geese: Entremuros r = 0.883, p<0.001, n = 60; Caracoles r = 0.736, p<0.001, n = 111; Cerro de los Ánsares r = 0.802, p<0.001, n = 20; purple gallinule: Entremuros r = 0.468, p<0.001, n = 91; Fig. 4). In Entremuros, four fecal samples from purple gallinule displayed higher Pb levels than predicted by the observed Al concentrations (Fig. 4b). Furthermore these samples had Pb>34 µg/g (d.w.), a level proposed as indicative of exposure to a point source of Pb pollution [32], [33] (i.e., such as Pb shot ingestion). Indeed, a better Pb-Al correlation was found for this species in Entremuros without these four samples (r = 0.619, p<0.001, n = 87). Finally, fecal samples with levels >34 µg/g showed significantly higher values of Pb/Al ratios than those with Pb levels lower than 34 µg/g (F_1,89_ = 89.68, p<0.001).

**Figure 5:**
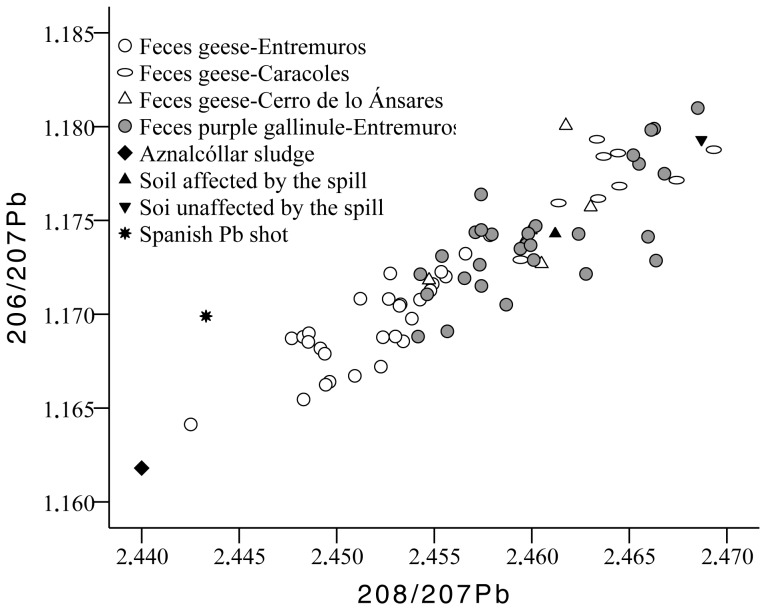
Relationship between Pb isotope ratios ^206^Pb/^207^Pb and ^208^Pb/^207^Pb in feces. Additionally, the figure shows mean Pb isotopic ratios described for the Aznalcóllar sludge, for Entremuros sediment affected by the spill, for unaffected sediment adjacent to the Entremuros [23], and for Spanish Pb shot [31].

**Figure 6:**
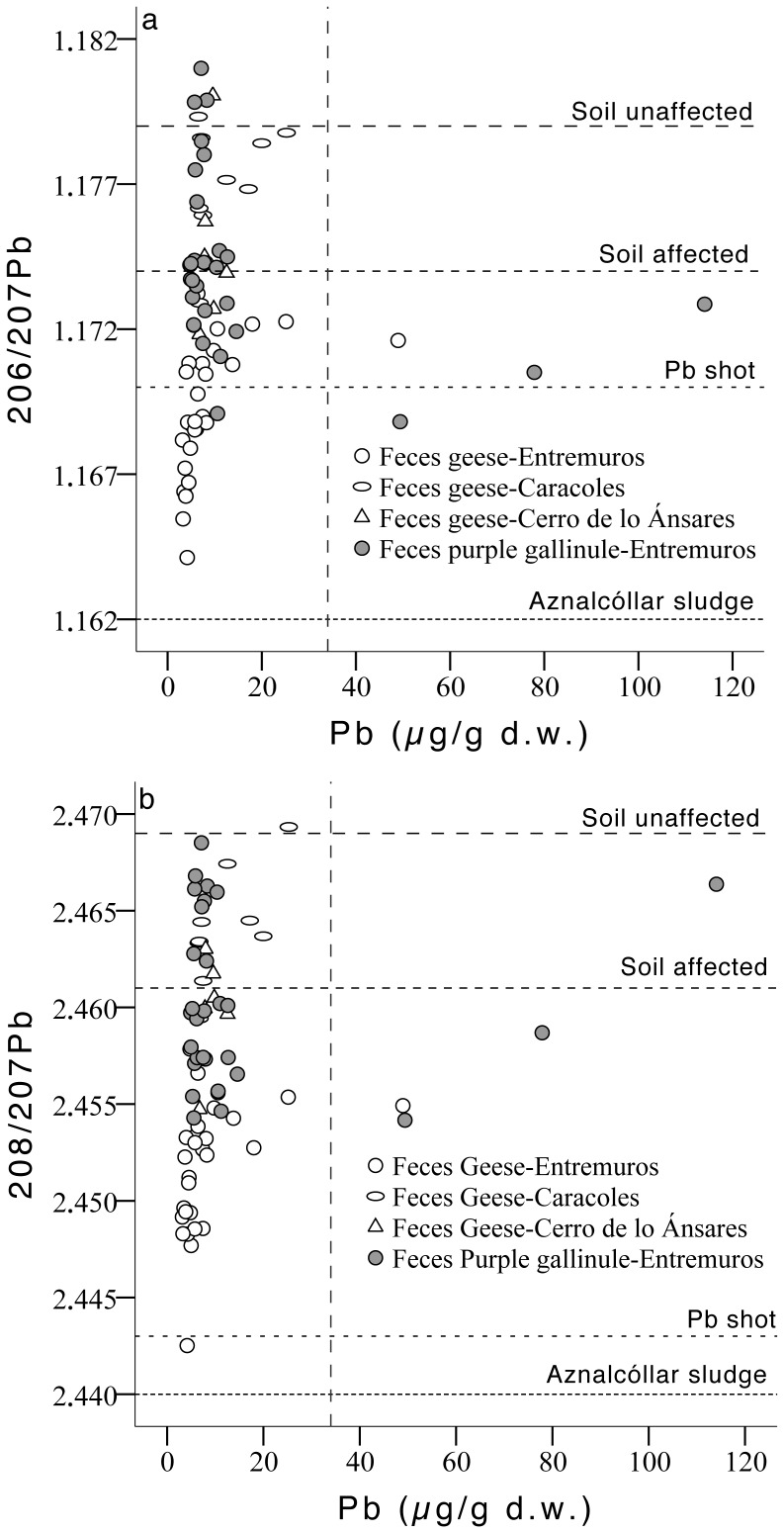
Relationship between fecal Pb concentrations and (a) ^206^Pb/^207^Pb isotope ratios, and (b) ^208^Pb/^207^Pb ratios for feces. The grey vertical dotted line shows the 34 μg/g (dry weight) Pb level. Values above this suggest exposure to point sources of Pb [32], [33]. Additionally, the figure shows mean Pb isotopic ratios described for the Aznalcóllar sludge material, for Entremuros sediment affected by the spill, for unaffected sediment adjacent to the Entremuros [23], and for Spanish Pb shot [31].

Differences in fecal mean isotopic ratios for ^206^Pb/^207^Pb and ^208^Pb/^207^Pb were detected between sites (F_2,68_ = 31.92, p<0.001; F_2,68_ = 42.46, p<0.001; respectively), and between species (F_1,68_ = 44.22, p<0.001; F_1,68_ = 75.27, p<0.001; respectively; Fig. 5). Fecal samples collected in Cerro de los Ánsares and Caracoles showed similar ^206^Pb/^207^Pb ratios, which were higher than those for Entremuros. For the ^208^Pb/^207^Pb ratio, a similar trend was detected, with similar values in samples collected in Caracoles and Cerro de los Ánsares, and lower values in Entremuros. However, in this case, the difference between Cerro de los Ánsares and Entremuros was only marginally significant (p = 0.05, Tukey test). For both ratios, lower mean values were detected in fecal samples from geese than in purple gallinule (Fig. 5). Fecal samples with Pb >34 µg/g also showed ^206^Pb/^207^Pb ratios compatible with those described for Spanish Pb shot pellets and sediment affected by the Aznalcóllar spill (Fig. 6a), while ^208^Pb/^207^Pb ratios for these high Pb samples were nearer those described for spill affected sediment (Fig. 6b).

**Figure 7:**
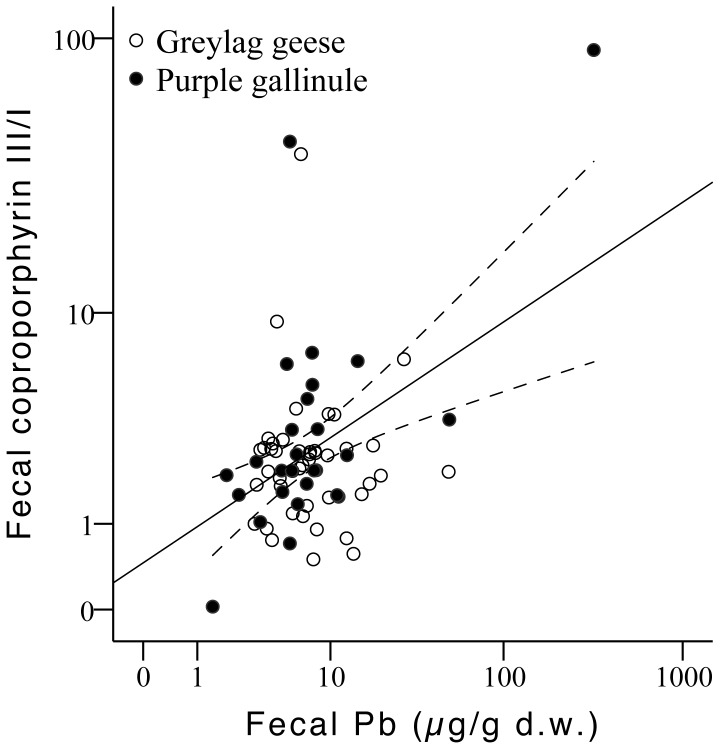
Relationship between Pb concentration and the coproporphyrin III/I ratio in fecal samples from greylag geese and purple gallinule collected in Doñana (r = 0.420 p<0.001, n = 71; independently for each species r = 0.007 p>0.05, n = 44; r = 0.636 p<0.001, n = 27; for greylag geese and purple gallinule, respectively).

Finally, the statistical analysis regarding biliverdin concentrations showed significant differences between species, with higher values in geese than in purple gallinule (F_1,66_ = 13.53, p<0.001; Table 2). Differences among sampling seasons were also observed (F_2,66_ = 8.04, p = 0.001), and a negative relationship was found with Pb concentrations (F_1,66_ = 9.50, p = 0.003). For geese a positive relationship was detected between fecal Pb and coproporphyrin I/biliverdin and coproporphyrin III/biliverdin ratios (geese: F_1,42_ = 6.08, p = 0.018; F_1,40_ = 7.38, p = 0.010). Although no differences in porphyrin levels were detected between sites, correlations showed that the positive relationship with fecal Pb was clearer in samples collected in the Entremuros (coproporphyrin I: r = 0.477, p = 0.009, n = 29; coproporphyrin III: r = 0.524, p = 0.004, n = 29). For purple gallinule, a positive relationship was detected between fecal Pb and coproporphyrin III/biliverdin ratio (F_1,23_ = 4.50, p = 0.045, r = 0.447, p = 0.019, n = 27), the coproporphyrin III/I ratio (F_1,25_ = 16.96, p<0.001, r = 0.636, p<0.001, n = 27; Fig. 7), and mesoporhyrin IX/biliverdin ratio (F_1,25_ = 15.13, p = 0.001, r = 0.614, p = 0.001, n = 27).

**Table 2:** Geometric mean (range) of porphyrins and biliverdin concentrations (nmol/g dry weight) in feces of greylag geese and purple gallinule collected at different sites in Doñana.

Species	Purple gallinule	Greylag geese		
Site	Entremuros	Entremuros	Caracoles	Cerro de los Ánsares
N	27	29	10	5
Coproporphyrin I	0.36 (nd†–2.55)	0.45 (0.01–1.47)	0.30 (nd–1.11)	0.51 (0.08–2.15)
Coproporphyrin III	0.91 (nd–3.76)	1.04 (0.05–3.44)	0.67 (0.24–1.70)	0.66 (0.19–3.17)
Mesoporphyrin IX	0.01 (nd–0.27)	0.03 (nd–0.80)	0.13 (nd–2.14)	0.01 (nd–0.55)
Protoporphyrin IX	0.25 (nd–0.68)	0.19 (0.01–1.09)	0.45 (0.23–1.20)	0.63 (0.37–1.13)
Biliverdin	14.15 (0.99–80.13)	46.97 (2.92–192.13)	25.83 (1.58–160.94)	45.79 (14.78–222.43)

† nd = below detection limit.

## Discussion

Ten years after the Aznalcóllar mine spill (1998–2008), this study shows that there had been a progressive decrease in exposure to Pb in greylag geese in various parts of the Doñana National and Natural Parks. In the Entremuros, the site most affected by the spill, the mean fecal Pb concentration in geese had decreased to 3 µg/g d.w. in 2005/2006, well below the 15 µg/g d.w. previously reported in the 2001/2002 wintering season [24]. The same trend was also detected in geese feces from Caracoles. Furthermore, the mean fecal Pb levels in Caracoles for geese (2.2 µg/g in 2007/2008) were similar to those reported for the 2001/2002 winter in areas unaffected by the spill, i.e., in the Cantarita rice fields (2.6 µg/g, a site bordering the Entremuros within the Natural Park), or Escupidera prairie (4.3 µg/g, a site within the National Park) [24]. Among the three sites studied here, geese feces from the Cerro de los Ánsares showed the highest mean Pb concentration (5.2 µg/g), and this was also higher than previously described for the same area (2.5 µg/g; [24]). However, overall, Pb concentrations in geese feces recorded here were within the range reported for waterfowl feces in uncontaminated areas (such as National Wildlife Refuges) in the USA, i.e., 10 µg/g d.w. [21], [25], [43], [44]. Also, fecal Pb levels detected here were within the range described in feces from hunted mallards (without Pb shot in their gizzards) from the Ebro Delta, Spain [32]. For purple gallinule in the Entremuros, although a slight increase in fecal Pb level was detected in the winter of 2007/2008, (which may reflect a change in diet or feeding area), mean Pb concentrations were still well below 10 µg/g d.w.

As expected, differing Pb isotope profiles were detected in the feces of geese from each site sampled. In Entremuros, the ^206^Pb/^207^Pb and ^208^Pb/^207^Pb isotopic ratios were confined between those described previously for the Aznalcóllar sludge and for sediment affected by the spill [23]. In contrast, the isotopic ratios for other samples were mostly confined between mean values described for sediment affected and unaffected by the spill [23]. Also, quite different isotopic profiles were detected in geese and purple gallinule feces from the Entremuros, which suggests a clear difference in Pb exposure pathways/sources between the two species. The reason behind this difference remains unclear, but these species are known to have quite different dietary preferences and may ingest with food items and/or retain in the gizzard markedly different amounts of sediment to aid the digestion of plant material in their gizzards (i.e., grit), but also incidentally when they feed. Geese are herbivorous, feeding mainly on *Scirpus* sp. and *Plantago* sp. in this area [24], [45], and tend to hold mean values between 10.8 and 28.7 g of grit in their gizzards [40], [46]. In contrast, gallinules are more omnivorous, feeding primarily on *Typha* sp. and seeds of *Cyperaceae* species (*Carex divisa, Scirpus* spp), but also non-plant material, and mineral matter can represent up to 63% of their gizzard contents [47].

For greylag geese, the mean Pb concentration in feces from the Entremuros was within the range expected when considering the average percentage of estimated ingested sediment (%EIS) described for this species in this area (8%; [24]). If we assume that the mean digestibility of plants on which geese feed at Entremuros is 37% [24], that all Pb ingested comes from sediment (and not plants), that it is poorly absorbed in the digestive tract (5%; [48]), and that the maximum Pb concentration in sediments from the Entremuros was 76 µg/g, then values up to ∼9 µg/g Pb would be expected in geese feces (calculated following the equations in [49]; i.e., Total % indigestible (feces) = 8%+((92%/100)*63%) = 65.96%; Expected feces Pb concentration = 76 µg/g/(65.96%/8%) = 9.2 µg/g). Thus, based on these results, the most common source of Pb exposure for geese seems to be the ingestion of slightly spill-contaminated sediment. In the case of purple gallinules, sediment ingestion can be high though, and gizzards with up to 63% mineral matter have been described in hunted individuals from the Guadalquivir marshes. This material was mainly coarse sand with a particle size up to 3 mm [47]. Results obtained here suggest that sediment ingestion may again represent a common source of Pb exposure in purple gallinule, i.e., the ^206^Pb/^207^Pb and ^208^Pb/^207^Pb isotopic ratios obtained showed values closely related to those previously described for sediment affected and unaffected by the spill. Furthermore, a strong relationship between fecal Pb and Al was also detected for this species.

Despite the importance of sediment ingestion as a Pb source, some samples analyzed in the Entremuros showed Pb levels far higher than expected via this exposure route alone. These samples fell well above/outside the expected Pb/Al regression line representing the Pb level expected due to sediment ingestion, and Pb concentrations were >34 µg/g d.w. This level suggests ingestion of point sources of Pb, such as Pb shot [32]. Indeed, the prevalence of Pb shot pellets in the gizzards of geese that were hunted or found dead was up to 10% and 28%, respectively, and in purple gallinules was up to 7 and 2%, respectively in the Guadalquivir marshes in past studies [41], [42], [47]. In the case of geese, a significant decrease in Pb shot ingestion in hunted/trapped geese wintering in Doñana was reported by 1999–2002 (after the ban on the use of Pb shot for hunting, and after partial removal of Pb shot in the Cerro de los Ánsares dune, conducted in 1999–2000; [42]). Furthermore, Pb shot was absent in gizzards of geese shot during 2002–2004 [24]. Despite this, Pb-poisoned wintering geese in Doñana were reported throughout between 1999 and 2004 [42]. Interestingly, although the ^206^Pb/^207^Pb isotopic ratio of these outlier samples lay between the ratio described for Pb shot in Spain and the Aznalcóllar spill contaminated sediment, this relationship was less clear for the ^208^Pb/^207^Pb ratio. Here, these outliers plotted more within the range for Entremuros sediment affected and unaffected by the spill (Fig. 6). In the Entremuros, iron plaques rich in As can develop on the roots of emergent macrophytes such as *Scirpus* sp. and *Typha* sp. [49], and these species are indeed commonly eaten by geese (mainly *Scirpus* sp.) and purple gallinule (both plant general) in Doñana [45], [47]. Iron oxide plaques can promote the geochemical accumulation of many metals/metalloids, including Pb [50] within/around roots, bulbs and rhizome tissues. Furthermore, Pb concentrations up to ∼300 µg/g, and a strong correlation between Pb and As levels have been documented in thoroughly washed *S. maritimus* roots from polluted areas within the Entremuros [51]. However, other studies have not detected particularly high accumulation in tubers tissue [52] or translocation to upper plant parts [17] of Pb or As in these species after the Aznalcóllar accident (significant Cd/Zn accumulation was however highlighted).

Given that the Pb isotopic profile in iron plaque associated with macrophyte roots is likely to be similar to that found in the surrounding sediment, this may be acting as a point source of Pb for the birds studied here which is nevertheless difficult to distinguish isotopically from the bulk sediment. As suggested by previous studies [49], iron plaque may act as an accumulation site and an important point source of toxic metals (such as As and Pb) for herbivorous waterbirds for an extended period after a pollution event (such as a mine spill). Another possibility is that ‘hotspots’ related to Pb contamination at a meso-scale still exist in spill affected sediments within the Entremuros [51].

Through the analysis of fecal porphyrins and biliverdin, we have also detected potential changes in heme synthesis related to Pb exposure in geese and purple gallinule in our study area. The positive relationships we found between Pb and coproporphyrin I/biliverdin and coproporphyrin III/biliverdin ratios in feces from geese, especially in those collected in the Entremuros, and with the ratios coproporphyrin III/biliverdin, coproporphyrin III/I and mesoporphyrin/biliverdin in feces from purple gallinule collected at the same site, all suggest that heme synthesis is being affected by Pb. Previous studies have reported a general increase in the levels of bile porphyrins in poisoned mallards, with the increase in coproporphyrin III being higher than in coproporphyrin I [38]. Further, a significant relationship between biliary and fecal (intestinal) coproporphyrin III/I ratio has been described in geese from Doñana shot after the Aznalcóllar spill; with this ratio also being significantly related with Pb concentrations in the intestinal contents [24]. Here we have also observed this positive relationship between Pb levels and the coproporphyrin III/I ratio in purple gallinules.

The inhibition of the enzyme ALAD is considered one of the most sensitive effects related to Pb exposure [35], [38]. A recent study [53] showed that blood Pb levels, below that assumed to be background exposure (i.e., 20 µg/dl), were still able to inhibit its activity. Additionally, in agreement with our results, Baos et al. [54] recently provided evidence of long-term, multigenerational consequences on white stork (*Ciconia ciconia*) due to the widespread low-level contamination left as a consequence of the Aznalcóllar spill. Furthermore, Baos et al. [55] suggested that birds exposed to sublethal Pb levels after the Aznalcóllar spill could be at risk through altered physiological responses linked to behavioral and metabolic processes necessary for survival. Similarly, 8 years after the spill, elevated accumulation of heavy metals has been described in reptiles from spill-affected areas [56]. Finally, we note that although the present work focused on Pb, the exposure to other toxic metals/metalloids (such as As or Cd) could also enhance these observed effects [24], [49].

Further work is now needed to understand the nature of the apparent point source of Pb pollution that is being recorded in the feces of some of these birds, and to pinpoint its exact source (be it Pb shot, metals accumulating on iron plaque or hot spots of polluted sediment). In this context, although the study of Pb isotopic profiles in feces appears to be an effective tool to help to differentiate between pollution sources the combined use of element ratios (such as Pb/Al or Fe/As) and perhaps other non-traditional stable isotope signatures may yet prove worthwhile [57].
