# Correction: Monitoring of Pb Exposure in Waterfowl Ten Years after a Mine Spill through the Use of Noninvasive Sampling

**DOI:** 10.1371/annotation/f7eb04d1-9032-49f4-b805-67c0342f4865

**Published:** 2013-08-13

**Authors:** Monica Martinez-Haro, Mark A. Taggart, Hugues Lefranc, Rosa C. Martín-Doimeadiós, Andy J. Green, Rafael Mateo

There were multiple errors and omissions in the Funding Statement. The correct Funding Statement is: MMH was supported by a project funded by the Consejería de Medio Ambiente, Junta de Andalucía, under a CSIC contract, and by a postdoctoral grant (SFRH/BPD/73890/2010) funded by the Portuguese Foundation for Science and Technology (FCT), the European Social Fund and POPH programme. Currently, MMH benefits from a Marie Curie Intra-European Fellowship for Career Development (PIEF-GA-2011-299747) within the 7th Framework Programme (FP7 2007-2013) of the European Commission. Study funded by MICINN (under CGL2007-62797). The funders had no role in study design, data collection and analysis, decision to publish, or preparation of the manuscript.

In addition, the affiliations listed for the first author are incorrect. Monica Martinez-Haro is affiliated neither with 1, Instituto de Investigación en Recursos Cinegéticos – IREC (CSIC-UCLM-JCCM), Ciudad Real, Spain, nor with 2, Department of Wetland Ecology, Estación Biológica de Doñana – EBD (CSIC), Sevilla, Spain, but with IMAR-Instituto do Mar, Department of Life Sciences, University of Coimbra, Coimbra, Portugal. 

